# Case report: First criminal conviction of dog fighting in Brazil: an international network organization

**DOI:** 10.3389/fvets.2023.1327436

**Published:** 2024-01-08

**Authors:** Esther Espejo, Alina Galante, Tália Missen Tremori, Louise Bach Kmetiuk, Matheus Araujo Laiola, Alexander Welker Biondo, Paulo Maiorka

**Affiliations:** ^1^Department of Pathology, Faculty of Veterinary Medicine and Animal Sciences, University of São Paulo, São Paulo, Brazil; ^2^Department of Veterinary MedicineFederal University of Paraná, Curitiba, Brazil; ^3^Department of Pathobiology, Purdue University, West Lafayette, IN, United States; ^4^Police Department for Environmental Protection, Curitiba, Brazil

**Keywords:** dog fighting, animal cruelty, forensics, international crime, pit bull

## Abstract

Although banned in several countries worldwide, dog fighting has remained a challenge, particularly on criminal investigation, recognition, and prosecution. Besides animal cruelty, dog fighting has been controlled mostly by criminal organizations and accompanied by illegal gambling and drug trafficking. While such competitions may be impaired by advances of legislation on animal welfare and media coverage, international organized crime has been migrating to less regulated and enforced countries. The case herein reported a flagrant dog fighting investigation in an international event involving 27 Pitbull dogs in Mairiporã, located 50 km outside São Paulo City, Brazil. An international network of dog fighting was revealed at the tournament, along with presence of organizers from USA, Mexico, and Peru. Proof was obtained on-site about other similar past and future competitions in other Latin American countries. Dogs were rescued, thoroughly examined for signs of animal cruelty, surveyed for potential diseases, and tested positive for visceral canine leishmaniasis. The process conducted by the state hearing resulted in the highest criminal sentence attributed to animal cruelty in Brazil to date, serving as jurisprudence for future prosecutions. Forensic veterinary medicine was essential in this case as a specialty for police and court assistance, leading to detailed and undeniable report of animal cruelty.

## 1 Introduction

Although dog fighting has been reported worldwide, few studies have focused on its legal consequences (1, 2). Considered as cultural and traditional activity, dog fighting has persisted as reminiscent of legal and social acceptance (1, 3, 4). Increasing public awareness on animal cruelty has gradually criminalized such abusive events, which brought more restricted laws and dog fighting ban in several countries (4, 5). Despite scarce reports and still undetermined acceptance and spreading, evidence has shown that dog fighting has continued nationally and internationally, mostly associated with organized crime (3, 6–8).

According to the Brazilian Criminal Code (Law 2,848, of September 7th 1940), article 158 “when violation may result in direct or indirect marks, body (*corpus delicti*) examination become indispensable, mandatory despite indictee’s confession” (9). Despite body examination must be thoroughly performed as proof of true facts in human forensics, animal cruelty cases have also required such legal procedure by analogy (10). Animal forensics, considered as exclusive activity of certified veterinarians, has been focused on meticulous description and analysis of lesions and behavior of animal victims, measuring cruelty nature and degree as basis for dosimetry of imposed penalties (11). Recognition of animal cruelty, neglection of veterinary attendance, use of controlled drugs, and diagnosis of infectious disease may also be considered as part of animal cruelty, professional ethics, and even public health crimes (5, 12, 13).

Although Brazil has no historical record of major competitions, clandestine dog fighting may have occurred nationwide as isolated events, involving few animals, with no legal evidence or appropriated animal forensics. In addition, recent studies in Brazil have shown increasing connection of dog fighting to street and former inmate gangs, organized in mafia groups, and developed as transnational organized crime controlling the market of illicit activities (14). In fact, dog fighting has been associated with other crime practices such as illegal drug and gun traffic, illegal gambling, and other violent criminal activities (3). In such a scenario, international associations organizing major events with participants of different nationalities may perpetuate dog fighting worldwide, based in countries with no or less prohibition, public disapproval, restrictions, and penalties (3).

This is the first case of legal conviction for dog fighting, besides the highest penalty imposed by animal cruelty in Brazil, surpassing the Brazilian animal serial killer case (11). In addition, this is the first report of dog fighting as an international source of zoonotic disease, as dogs had international transit documents.

## 2 Case description

In December, 2019 at the city of Mairiporã, about 50 km (31 miles) far from São Paulo city, an illegal ongoing Pitbull dog fighting event called “International Circuit 4 × 4” was caught by a taskforce comprised by Civil Police officers of São Paulo and Paraná States. In total, 40 men were caught in-place carrying out animal cruelty and illegal betting, including two minors whose father fled the scene. The organizer was arrested for criminal association, animal cruelty resulting in death, illegal betting in public place, and recruitment of minors.

The event was arranged in 13 dog combats, with a total of 19 Pitbull dogs found alive and rescued. Another 10 Pitbulls were found dead, including one being cooked in a barbecue grill and freely offered to participants, and the other nine slaughtered in pieces into freezers, with fragments offered as food for competition dogs. Additionally, as unfold investigation, eight Pitbulls were rescued from an illegal kennel located in Itu City, 97 km (60 miles) far from Mairiporã City.

**Figure 1:**
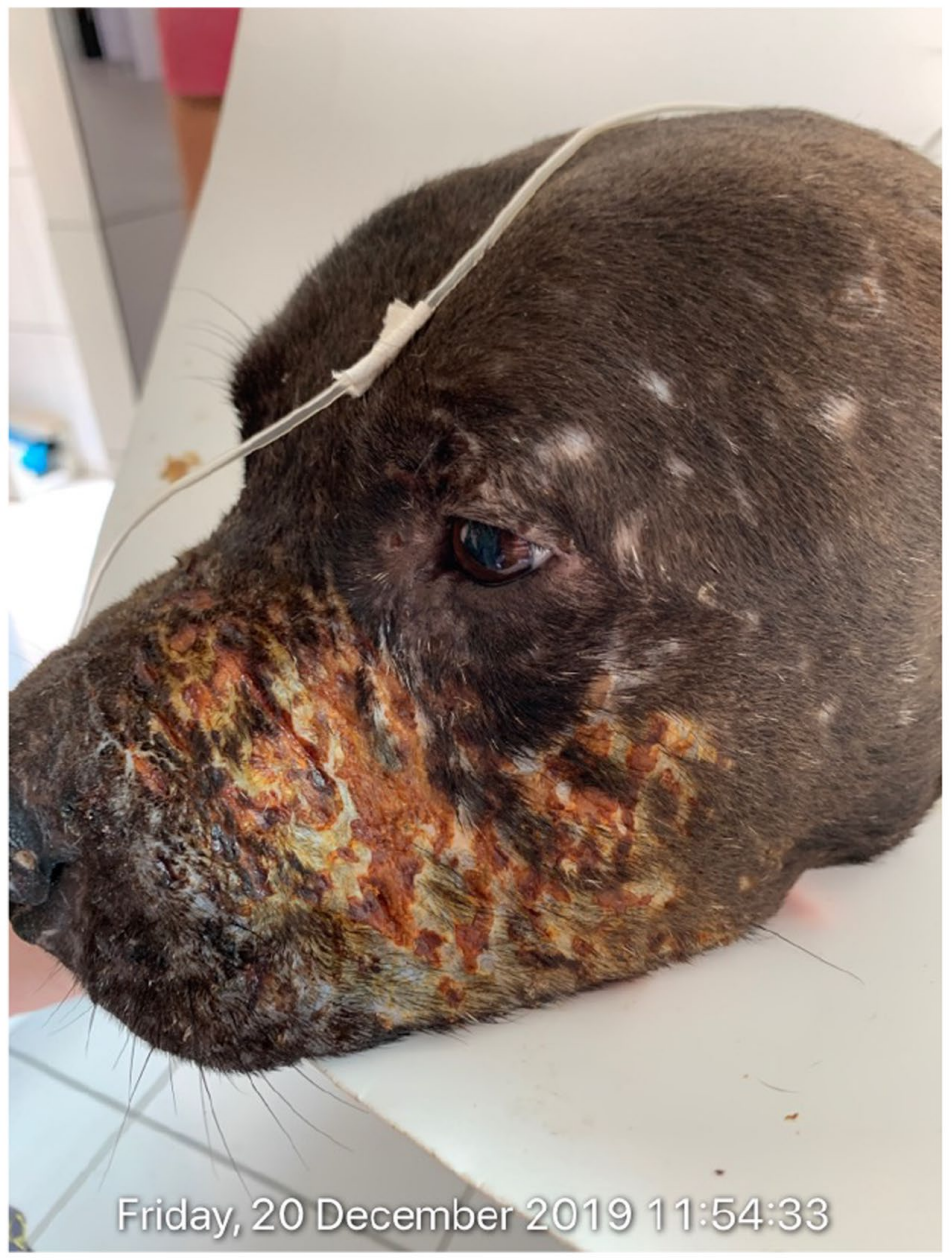
Body exam (*corpus delicti*) during veterinary forensics of Hades, one rescued Pitbull dog, which presented several lesions on left head side, some self-healing and some not, maybe due to lack of veterinarian assistance and frequent fighting. Dog died few hours later at the same day of forensics, due to advanced cruelty injuries.

**Figure 2:**
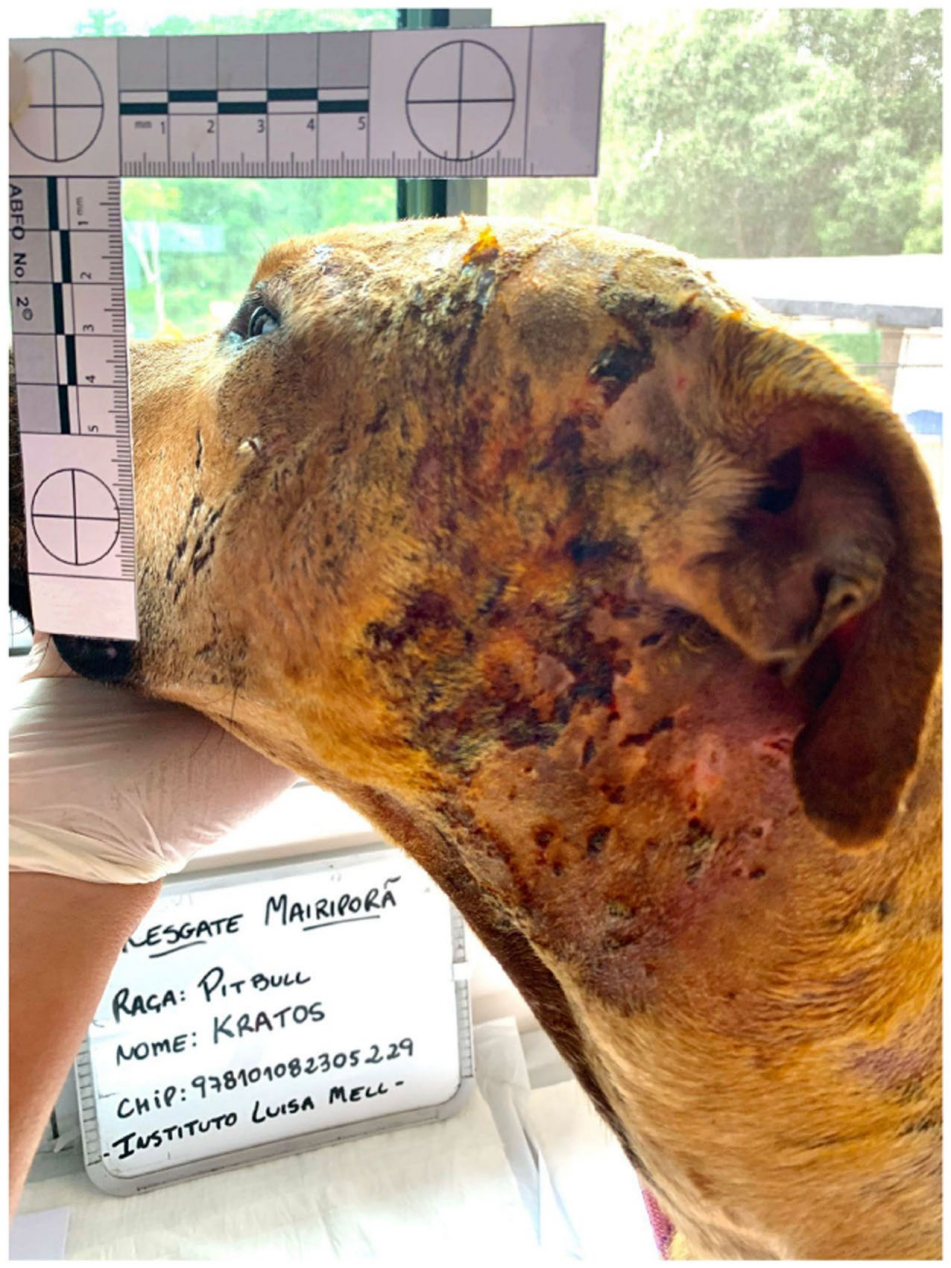
Body exam (*corpus delicti*) during veterinary forensics of Kratos, one rescued Pitbull dog, showing wounds, lesions, and punctures due to bites throughout the head.

**Figure 3:**
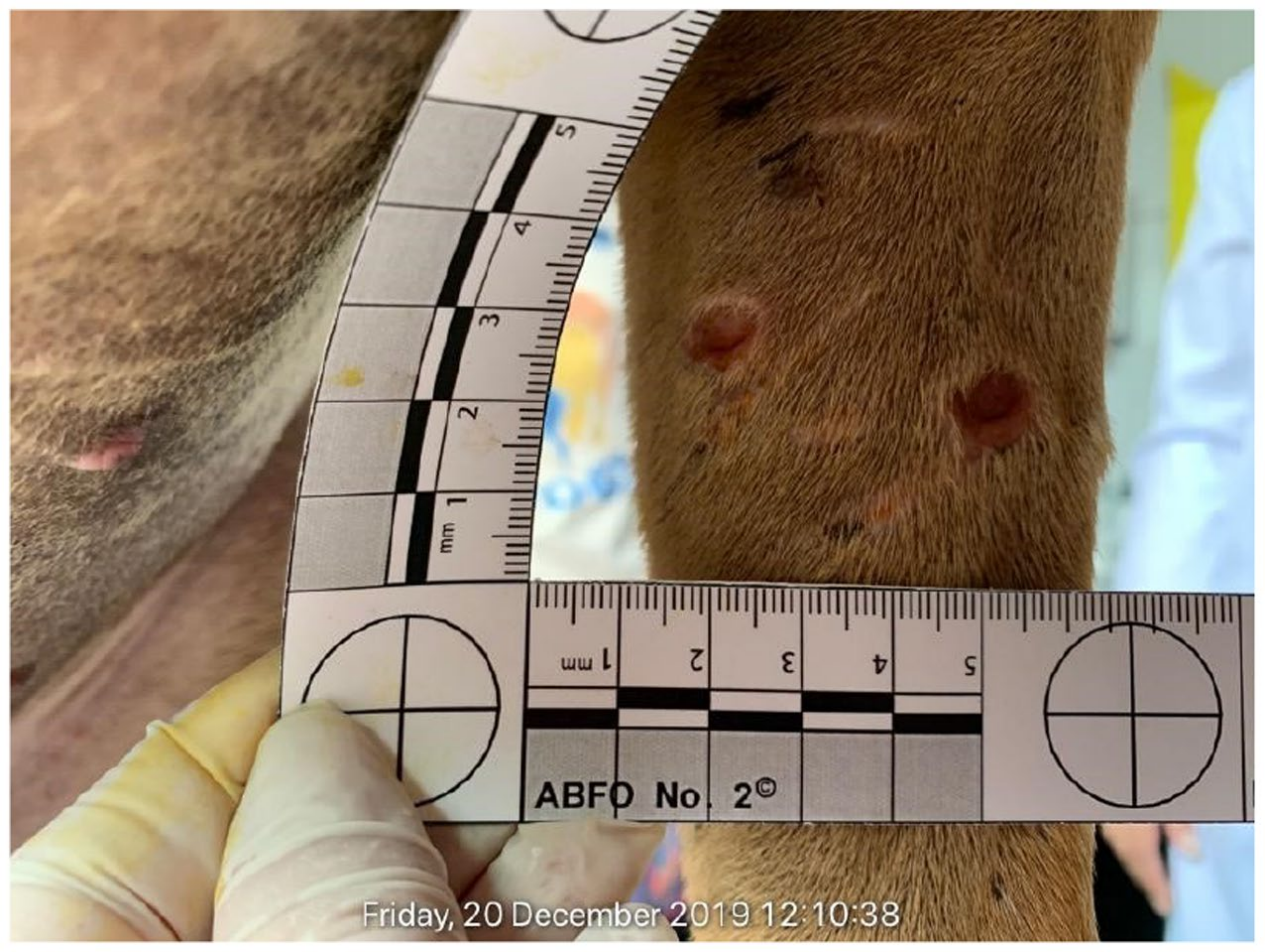
The body exam of Kratos has also shown bite punctures on the left thoracic limb.

Of the 27 examined dogs, 17 rescued from the fighting event in Mairiporã city presented injuries as shown (Figures 1–3), with serious recent injuries along to old deep scars, likely due to bites and other aggressions during combats, with six/27 (22.2%) seropositive dogs for canine visceral leishmaniasis. Another 10 dogs owned by the same organizer were found in Itu city and presented few or no injuries, probably as they were at training stage for future fighting. In addition, two more dogs were rescued from Mairiporã city and died due to critical health status and intensity of injuries, before the clinical examination by legal veterinarian expert.

According to the final sentence by the São Paulo State Court, criminal materiality and authorship were fully witnessed, documented, and proved beyond reasonable doubt. Forensic results for living dogs and necropsies of dead dogs were also documented and presented in the forensic report. The hearing testimony of expert witnesses EE and PM, both co-authors, was considered crucial for animal cruelty recognition during trial, unprecedented in Brazilian justice (15). Numerous photographs and videos were taken and analyzed from the crime scene, clinical evaluation and also images from the animals’ necropsy. Forensic Report was composed of 227 pages and 431 photographs used to illustrate lesions that were all described according to the veterinary forensic standards used for animals in Brazil (10, 11).

The criminal proceedings unusually lasted 42 months due to the COVID-19 pandemic restrictions. All three hearings during the criminal proceedings were remotely performed, with the two Experts questioned by the prosecution and defense, clarifying the Expert Report descriptions and answering additional questions on suffering and health status of dog survivors (10, 11, 15).

Conviction sentences varied from 6 months and 22 days of simple confinement to 5 years and 3 months of prison, and to 31 years and 14 days of detention. In addition, convicts were charged with 1,216 fine-days, loss of all apprehended materials and values, and the remaining dogs. The three sentences for the crimes committed referred only to the event organizer. The judicial process regarding the other 40 men who were caught in the act was separated from the main process to individualize sentences and remains in ongoing legal secret at this point. Fine details have been kept in judicial secrecy and legal circumstances, so only basic information could be inserted to this point.

## 3 Discussion

Dog fighting has evolved from its origin of traditional local and amateur events to current association with international crime organizations worldwide. Fighting dogs have been historically used for fighting by street gangs as weapons of status, intimidation, and aggression, as dogs may be trained to attack humans (3, 16). A series of other crimes have been directly associated with dog fighting, particularly illegal gambling, gun smuggling and drug traffic (3, 4, 8, 17). Dog fighting supply itself may be associated with animal cruelty including illegal breeding, raising, trading, and training (5, 16). Finally, attendees, members, subscribers, and followers of dog fighting events have been considered as criminal supporters, accomplices, and unlawful individuals, particularly when children and youth are intentionally exposed to ongoing violence and animal cruelty (3, 18).

Dog fighting may also be considered a distinct and organized form of subcultural crime, according to Green Criminology perspective (3). As several crimes against animals may escape general classic criminology scope, such narrow-minded approach may fail to recognize animal abuse and cruelty connection to other violent crimes against life (3). Recent studies on organized crime in Brazil have shown continuous growing of incarcerated gangs, linked to transnational mafias, and operating in a wide range of violent crime categories (14). Thus, advanced societies have demanded criminalization and enforcement of such cruel and painful actions involving animals, along with illicit gambling, gun trade and drug traffic (3, 8).

Criminal investigations of dog fighting worldwide may be very complex, requiring special techniques such as undercover policemen infiltrating into gangs and continuing surveillance for long periods (6). Secret operations and deep financial investigations may also be necessary in order to monitor ongoing criminal activities (8). In the United Kingdom, dog fighting has been reportedly organized by urban criminals who mostly conducted such crimes in enclosed rural environments, with no access to authorities (1, 3). In addition, as other crimes, dog fighting has migrated its organizations to internet platforms, in a new cybernetic crime type and requiring multi-professional taskforce for efficient investigation (6, 8). Besides promoting events, illegal websites have also provided opportunities for publications and posts on dog fighting, from selling dog litters to breeding with winner dogs (1, 3).

Besides animal cruelty as consequence of physical wounds and psychological traumas, dog fighting has also been associated to criminal activities such as illegal gambling and drug trafficking (19). In addition, animal cruelty has been considered a predictor and associated with human cruelty including domestic violence, child and elderly abuse, bullying, and juvenile delinquency. In such a scenario, children attending or being raised in a dog fighting environment may become violent adults due to behavioral impact (19) Thus, strong evidence has established a link between animal cruelty and human violence, in which “when animals are abused, people are at risk; when people are abused, animals are at risk” (20).

Additionally, association of mafia, gang or criminal groups with dog fighting may be difficult to prove as it may rely on appropriate investigation and surveillance (4, 6). Current Brazilian laws consider acts leading to pain, injury, and mutilation as animal abusive and cruelty, subject to criminal and civil prosecution (10, 11). Even so, practitioners, consumers and other supporters have inherited, passed, and perpetuated such animal cruelty, particularly through internet (3, 7).

Animal welfare has been completely ignored in dog fighting, with dogs constantly exposed to neglect, chronic stress, suffering and cruelty. Malnutrition, stress, and inappropriate drug administration (such as hormones) may cause immunosuppression, animal health deterioration and vitality impairment (5). In such a scenario, opportunistic and zoonotic infections such as leishmaniasis may be favored and more easily transmitted and spread. Even when rescued and clinically recovered, dogs have mostly acquired behavioral disorders that can be hard to put up for future adoption.

Veterinary forensics has been crucial herein and in other abusive events involving animals, as effective prosecution and conviction often rely on forensic evidence and documentation as proof of animal cruelty and lack of veterinary assistance (10, 11). In addition, animal forensics has been useful to pinpoint and describe the animal status in legal details including clinical, psychological, and behavioral conditions (17, 18). Moreover, seropositive fighting dogs for visceral canine leishmaniasis have indicated real transmission risk, particularly by dog breeding, blood contact in dog-to-dog during fights and dog-to-human during handling (12, 13). Dogs are the main host of *Leishmania infantum*, which is mainly transmitted by sandfly (21). A total of 53,715 human VL cases were reported nationwide, mostly occurring in northeastern, southeastern, and midwestern Brazil (22).

On top of that, participants in the present report slaughtered and consumed dog meat, which may be an uncommon but possible way of *Leishmania* infection. Finally, fighting dog meat may also contain anabolic steroids and other harmful drugs.

At the clinical examination, four/19 (21.0%) dogs presented chronic pulmonary problems likely due to daily direct exposure to weather conditions, mostly short chain-leashed to small outdoors woody kennels. Also, dogs mostly presented abrasive and excoriating dermatological lesions on the neck nine/19 (47,3%), a likely result of daily heavy metal collar, along with scars in different healing stages 19/19 (100%), probably from previous combats. In addition, other similar lesions reported in frequent dog fighting were found including fractured teeth and periodontal disease ten/19 (52,6%), pyometra two/19 (10,5%), bloody diarrhea and gastrointestinal diseases four/19 (21%) (16, 18, 23).

Although owners argued that dogs were not incited to fight and “expressed their natural behavior,” animals likely underwent intensive training, leashing isolation, and gameness (non-stop “finish-job”) fighting, regardless of their chronic severe injuries (4). All dogs herein demonstrated submissive behavior and fear towards inspectors, and aggressiveness to other dogs.

The police incident report at the time also described commercial androgenic steroids and testosterone found at the scene, along with syringes and needles, which may explain the observed damaging effects such as seborrhea, hydric retention, infertility, prostatic hyperplasia, aggressiveness, and violent behavior disorders observed herein. In addition, policemen found trophies, collars, harnesses, ropes, laptops, cell phones, a firearm with several intact ammunitions, t-shirts with event logo and dog fighting advertisement, bank envelopes, and cash of Brazilian reals (U$7,359.42), USA dollars (U$4,760.00), Peruvian soles (U$121.05), and Mexican pesos (U$206.08).

A competition referee of USA citizenship was identified and arrested in flagrant crime, along with three trainers and handlers of Peruvian and Mexican citizenship. Although indicted for animal cruelty and other crimes, all foreigners flew out of Brazil just after the first hearing and bond release, leaving behind their apprehended Pitbulls and materials. An upcoming event was already scheduled and would be held in Honduras, Central America.

Investigation and high media exposure have stimulated other reports of animal cruelty, leading to apprehensions of fighting dogs in other cities of São Paulo, Paraná, and Minas Gerais States (24). In November, 2022 a policeman was sentenced to 11 years and 9 months of prison due to animal cruelty and neglect during an fighting dog kennel inspection (25).

Although Brazil has required an International Veterinarian Certificate on dog health status issued by the veterinary authority from the exporting country, no restriction has been made on dog breed entry to date. Meanwhile, Pitbulls and other fighting breeds have been banned in several worldwide countries such as Australia, Colombia, Croatia, Denmark, France, Germany, Hong Kong, Ireland, Israel, Malaysia, New Zealand, Norway, Portugal, Puerto Rico, Qatar, Romania, Singapore, Spain, Switzerland, Thailand, United Arab Emirates and United Kingdom (26). In addition, dog fighting has been considered illegal in most South American countries, USA, Canada, South Africa, Australia, Japan, India, Pakistan, and Afghanistan (27). Dog fighting has been categorized as a crime since 2008 in all 50 USA states and in the District of Columbia, Guam, Puerto Rico, and US Virgin Islands (27). Although laws and penalties widely vary, most USA states consider illegal the dog possession for fighting purposes, and witnessing dog fighting either as gambler, breeder, host, or spectator (28). In the United Kingdom, dogfighting was considered a bloody sport and officially banned in 1835, with introduction of animal welfare laws.

Despite no specific laws in Brazil, dog fighting may be interpreted as animal cruelty practicing, and organizers, (Pitbull) breeders and event spectators may be prosecuted according to the Law 9605/1998 of environmental crimes (29). Although considered generic and insufficient for proper animal cruelty recognition, the law established criminal sanctions for anyone committing acts of abuse or mistreatment leading to injure or mutilation of wild, domestic, or domesticated, native, or exotic animals (Article 33). Even so, dog fighting was not addressed as crime by the Brazilian Justice system until the report herein, which brought widespread media coverage and ultimately led to criminal conviction. Only recently, the Federal Law 14,064/2020 was sanctioned, increasing details and penalties for abuse and cruelty of dogs or cats (30).

Federal Laws in Brazil have not specified dog fighting as cruelty crime. Despite not a law, the Resolution 1236/2018 issued by the Brazilian Federal Council of Veterinary Medicine finally defined and characterized several types of cruelty, abuse, and mistreatment against vertebrate animals (31). This Resolution has been used since by veterinarian experts in establishing the level of animal cruelty and suffering. As mentioned, the recent Federal Law 14,064/2020 increased the variety and penalties for companion animal abuse and cruelty.

Dogs bred for fighting do not only suffer during the events but may be constantly exposed to painful stimuli before, during, and after dog fighting occasions. In overall, dog fighting has been characterized by a sequence of active and passive animal abuse actions that can cause mild to severe psychological and physical injuries, including premature or sudden death (19). Moreover, dog fighters routinely undergo harmful procedures during trainings and prior to fighting, such as ear and tail removal to prevent fighting disadvantage, thus encouraging neck and limb bites, which may be hard to recognize as physical aggression and injury. Fighting dogs may also undergo teeth procedures to make them more dangerous to opponents; and given legal and illegal drugs to enhance fighting physique and aggression (19). Thus, dogs may suffer not only during the events, but subjected from birth to cruel training, poor care and constant stress mentally and physically affecting animal health and welfare (5).

No restriction on dog breed entry has been in effect to date in Brazil. Despite Pitbull and other fighting breeds have been banned in several countries. Thus, Brazil should firstly subscribe to international banning of fighting and aggressive dog breeds, training police officers and veterinarians at airports, ports, and land borders for accurate recognition of dog fighting breeds, along with adequate clinical examination for evidence of typical injuries, dog behavior and use, and proper assessment of motivation for entry and length of staying. As such control may not fully prevent inland breeding and dog fighting, the Brazilian Federal Council of Veterinary Medicine should issue a continuing training and nationwide protocol for clinicians on detection, recognition, and systematic notification of suspicious injuries related to dog fighting.

The judicial process conducted by the state hearing herein resulted in the highest criminal sentence attributed to animal cruelty in Brazil to date, serving as jurisprudence for future prosecutions in Brazil and abroad. Soon after the case of serial killer of animals in Brazil (11) the widespread media exposure increased awareness of population and legislators about animal abuse and a new law was approved increasing prison time for crimes of cruelty against dogs and cats. In the present case, there was an increase in complaints and investigations in Pitbull kennels in other states in the country (24, 25). Forensic veterinary medicine was essential as a specialty for police and court assistance, leading to detailed and undeniable descriptions of animal cruelty. As preventive measures, fighting dog breeds should be banned, restricted and/or monitored (microchipping) in Brazil and worldwide, ending such cruel use of animals for profit and entertainment.

## Data availability statement

The original contributions presented in the study are included in the article/supplementary material, further inquiries can be directed to the corresponding author.

## Ethics statement

The animal study was approved by the Animal Use Ethics Committee (protocol number 3650310821) of University of São Paulo. The study was conducted in accordance with the local legislation and institutional requirements.

## Author contributions

EE: Writing – original draft, Writing – review & editing. AG: Writing – review & editing. TT: Writing – review & editing. LK: Writing – review & editing. ML: Writing – review & editing. AB: Writing – review & editing. PM: Writing – original draft, Writing – review & editing.
